# CanRisk-Prostate: A Comprehensive, Externally Validated Risk Model for the Prediction of Future Prostate Cancer

**DOI:** 10.1200/JCO.22.01453

**Published:** 2022-12-09

**Authors:** Tommy Nyberg, Mark N. Brook, Lorenzo Ficorella, Andrew Lee, Joe Dennis, Xin Yang, Naomi Wilcox, Tokhir Dadaev, Koveela Govindasami, Michael Lush, Goska Leslie, Artitaya Lophatananon, Kenneth Muir, Elizabeth Bancroft, Douglas F. Easton, Marc Tischkowitz, Zsofia Kote-Jarai, Rosalind Eeles, Antonis C. Antoniou

**Affiliations:** ^1^ Centre for Cancer Genetic Epidemiology, Department of Public Health and Primary Care, University of Cambridge, Cambridge, United Kingdom; ^2^ MRC Biostatistics Unit, University of Cambridge, Cambridge, United Kingdom; ^3^ Oncogenetics Team, Division of Genetics and Epidemiology, The Institute of Cancer Research, London, United Kingdom; ^4^ Division of Population Health, Health Services Research and Primary Care, School of Health Sciences, Faculty of Biology, Medicine and Health, The University of Manchester, Manchester, United Kingdom; ^5^ Cancer Genetics Unit, The Royal Marsden NHS Foundation Trust, London, United Kingdom; ^6^ Department of Medical Genetics, University of Cambridge, Cambridge, United Kingdom

## Abstract

Prostate cancer (PCa) is highly heritable. No validated PCa risk model currently exists. We therefore sought to develop a genetic risk model that can provide personalized predicted PCa risks on the basis of known moderate- to high-risk pathogenic variants, low-risk common genetic variants, and explicit cancer family history, and to externally validate the model in an independent prospective cohort.

**MATERIALS AND METHODS:**

We developed a risk model using a kin-cohort comprising individuals from 16,633 PCa families ascertained in the United Kingdom from 1993 to 2017 from the UK Genetic Prostate Cancer Study, and complex segregation analysis adjusting for ascertainment. The model was externally validated in 170,850 unaffected men (7,624 incident PCas) recruited from 2006 to 2010 to the independent UK Biobank prospective cohort study.

**RESULTS:**

The most parsimonious model included the effects of pathogenic variants in *BRCA2*, *HOXB13*, and *BRCA1*, and a polygenic score on the basis of 268 common low-risk variants. Residual familial risk was modeled by a hypothetical recessively inherited variant and a polygenic component whose standard deviation decreased log-linearly with age. The model predicted familial risks that were consistent with those reported in previous observational studies. In the validation cohort, the model discriminated well between unaffected men and men with incident PCas within 5 years (C-index, 0.790; 95% CI, 0.783 to 0.797) and 10 years (C-index, 0.772; 95% CI, 0.768 to 0.777). The 50% of men with highest predicted risks captured 86.3% of PCa cases within 10 years.

**CONCLUSION:**

To our knowledge, this is the first validated risk model offering personalized PCa risks. The model will assist in counseling men concerned about their risk and can facilitate future risk-stratified population screening approaches.

## INTRODUCTION

Prostate cancer (PCa) exhibits marked familial aggregation and has one of the highest heritabilities of any common cancer.^1-4^ This is explained in part by rare pathogenic variants (PVs) in *BRCA2, HOXB13*, and possibly *BRCA1*, which are associated with moderate-to-high PCa risks,^5-14^ together with several hundred commoner variants conferring lower risks, identified through genome-wide association studies.^15-18^

**CONTEXT**

**Key Objective**
Can a genetic risk model that uses information on all known high-, moderate- and low-risk prostate cancer genetic susceptibility variants, together with residual cancer family history (FH) information, accurately predict men's risk of developing prostate cancer in the future?
**Knowledge Generated**
We developed a genetic risk model using data from 16,633 prostate cancer families. The model uses data on rare pathogenic variants in the moderate- to high-risk genes *BRCA2*, *HOXB13*, and *BRCA1*, a polygenic score on the basis of 268 common low-risk variants, and detailed cancer FH to predict the future risks. The risk model predicted incident prostate cancers in an independent cohort of 170,850 prospectively followed men with high discrimination and good calibration. The majority, 86%, of incident prostate cancers occurred among the half of men with the highest predicted risks.
**Relevance**
This multifactorial risk prediction model is inclusive of genetic variant data and FH information and will be beneficial for counseling of men in cancer family clinics, and guide future research evaluating risk-stratified population screening approaches.

Men currently seen in family or genetics clinics are counseled on the basis of descriptive family history (FH) and ethnicity-specific risk estimates^19,20^ and/or average PV risk estimates.^20-22^ However, risks for *BRCA1*/*2* and *HOXB13* PV carriers have been found to vary by PCa FH.^9,10^ In addition, polygenic scores (PGS) on the basis of common variants can provide considerable risk stratification,^18,23-25^ in the general population and in men with FH,^23^ *BRCA1*/*2*,^26-28^ or *HOXB13* PVs.^13,27^ A comprehensive risk model, incorporating the joint effects of known and unknown genetic factors, should therefore provide better risk stratification and hence a more rational basis for counseling. Such models are now in widespread use in the management of breast and ovarian cancer risk.^29-33^ A PCa model would address similar clinical needs. Some genetic PCa risk models exist,^34-41^ but none combine data on detailed FH, PVs, and the latest PGS. None have been externally validated.

To support improved and consistent counseling of at-risk men on the basis of personalised future PCa risks, and to enable risk-stratified interventions, we developed a risk model on the basis of data from a large kin-cohort study and validated the model in an independent prospective cohort.

## MATERIALS AND METHODS

### Study Participants: UKGPCS

The UK Genetic Prostate Cancer Study (UKGPCS)^42^ recruited individuals with histologically confirmed PCa in three arms: a population-based arm that recruited men independent of age or FH, and arms enriched for young-age-at-onset PCa or PCa FH. Self-reported cancer FH data were collected through a questionnaire. We used data on the families of 16,633 European ancestry probands recruited from 1993 to 2017. Subsets had data available on *HOXB13* G84E (n = 11,500),^10,13,43^ *BRCA1* (n = 2,148), *BRCA2* PVs (n = 3,077),^44,45^ and a 268-SNP PGS (n = 11,149; Data Supplement [online only]).^18^

### Population Controls

To estimate the PGS population-distribution, we included 4,319 controls genotyped using the same SNP array as the cases, from (1) men without PCa personal or FH recruited through UKGPCS participating clinics, and (2) ProtecT trial participants with PSA < 0.5 ng/mL.^45,46^

### Study Participants: UK Biobank

The model was externally validated in UK Biobank,^47^ a prospective cohort study of volunteers recruited from 2006 to 2010. Data were available on 170,850 White British male participants without any cancer at recruitment (except nonmelanoma skin cancers). Participants provided baseline cancer FH information and were followed up prospectively through linkage with national registries. Data were available on a modified 268-SNP PGS and on the *HOXB13* G84E variant for all participants,^14,48^ and on *BRCA1*/*2* protein-truncating variants for 40% of the participants (Data Supplement).^49,50^

### Descriptive Familial Relative Risks

To explore familial aggregation patters in UKGPCS families, we estimated familial relative risks (FRRs) to relatives of the probands (Data Supplement).

### Risk Model Development

We used complex segregation analysis to fit genetic models for the observed cancer inheritance patterns in UKGPCS families.^51^ PCa incidence was assumed to depend on *BRCA2*, *HOXB13*, and *BRCA1* PVs, together with a polygenic component (PGC) to model residual familial risk. The PGC was assumed normally distributed, reflecting the combined effects of a large number of low-risk alleles. Additional models were considered, which allowed for a fourth hypothetical major gene following recessive, dominant, or multiplicative models of Mendelian inheritance. The average age-specific incidences across all genotypes and polygenotypes were constrained to agree with calendar period– and birth cohort–specific population incidences.^29,30,52,53^ Female relatives were assumed to be at risk of breast and ovarian cancer, following a similar model but without PGC. The models were parametrized by logit-transformed allele frequencies and log-relative risks (RRs) for genetic components; the log-standard deviation (SD) of PGC, which was assumed constant or age-dependent; and the logit-transformed proportion of the PGC that was explained by the PGS. Parameters were estimated by maximizing the joint likelihoods of the family members' phenotypes under the assumed genetic model, using MENDEL software (version 3.3).^54^ We adjusted for the nonrandom ascertainment of families by conditioning on data that may have influenced the ascertainment.^55^ The fit of different models were compared using the Akaike information criterion and likelihood ratio tests (Data Supplement).

### Known Genetic Components

For *BRCA2* and *BRCA1*, given the small number of carriers in UKGPCS, we assumed external estimates of age-specific RRs of PCa,^5^ breast and ovarian cancer, and allele frequencies.^29-31,33^ *HOXB13* G84E frequencies and RRs were estimated based on the data set. Guided by a previous study, we assumed a multiplicative per-allele effect, with birth cohort–specific RRs (born < 1930/≥ 1930).^10^

We used the best-fitting model to include a PCa PGS on the basis of 268 SNPs.^18,56^ We decomposed the PGC into one part explained by the PGS and an independent residual part explained by unidentified genetic effects,^31,33^ and estimated the fraction of the PGC explained by the PGS as a model parameter.

Guided by observations that FH is associated with higher PCa risk also for PV carriers,^9,10^ and that PGSs modify the risk for PV carriers,^13,26-28^ we assumed that the joint effects of PGC, PGS, and PVs on PCa risk are multiplicative.

### Sensitivity Analyses

We assessed the effect of the ascertainment adjustment on the basis of the method of PCa diagnosis (symptomatic, PSA testing, or unknown), and refitted the model in subgroups (Data Supplement).

### Model-Predicted Risks

We compared age-specific model-predicted FRRs with FRRs reported in observational studies.^1^ The model was used to estimate absolute PCa risks in example scenarios (Data Supplement).

### External Validation

We predicted 5- and 10-year prospective risks of developing PCa for the UK Biobank participants, using the data on age and FH available at baseline, PVs, and PGS. Only *BRCA2* and *BRCA1* protein-truncating variants were available, and hence, *BRCA1*/*2* PVs did not include pathogenic missense variants or large rearrangements; therefore, we assumed testing sensitivities of 83% for *BRCA2* and 65% for *BRCA1*. We compared the predicted and observed risks of PCa diagnosis, and assessed the model discriminatory ability and calibration (Data Supplement). We also assessed the model sensitivity and specificity at different quantiles of the risk distribution.

### Ethics

All participants provided written informed consent. UKGPCS was approved by the London Central Research Ethics Committee. UK Biobank was approved by the North West Multi-Centre Research Ethics Committee.

## RESULTS

The Data Supplement details the inclusion and the characteristics of the UKGPCS probands and their relatives. Thirty percent reported at least one PCa diagnosis in first-degree relatives (FDRs) or second-degree relatives. Fifty percent were diagnosed by clinical symptoms, 24% by PSA screening, and for 26% the method of detection was unknown.

The descriptive PCa FRR was 3.18 (95% CI, 2.92 to 3.45) for male FDRs in the population-based families. The FRRs were higher for brothers than fathers, and for FDRs of men diagnosed through PSA testing than for FDRs of men diagnosed through clinical symptoms (Data Supplement).

### Model Development

A detailed description of the model-fitting process is available in the Data Supplement. The most parsimonious model is summarized in Table 1, and included the effects of *BRCA2*, *HOXB13*, and *BRCA1*, together with a hypothetical recessively inherited allele and a PGC with age-dependent SD. The SD was 2.13 (95% CI, 2.00 to 2.27) at age 70 years and decreased at a relative rate of 0.989 (95% CI, 0.985 to 0.994) per year of age. The PGS explained 52.3% (95% CI, 50.3 to 54.4) of the polygenic SD. The predicted age-specific FRRs were consistent with previously published FRR estimates (Data Supplement).^1^

**TABLE 1.**
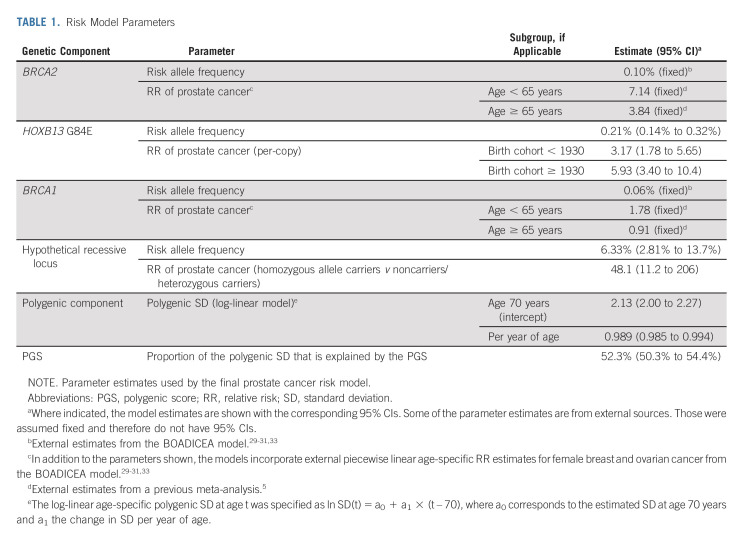
Risk Model Parameters

### Sensitivity Analyses

Ignoring the method of PCa detection in the ascertainment adjustment had a marked effect on the model parameters (Data Supplement), but resulted in model-predicted FRRs that were considerably higher and inconsistent to those reported in large epidemiologic studies (Data Supplement).^1^ This was driven by the subgroup of families ascertained through PSA-screened probands (Data Supplement). We therefore did not pursue these models further.

### Model-Predicted Absolute Risks

**FIG 1.**
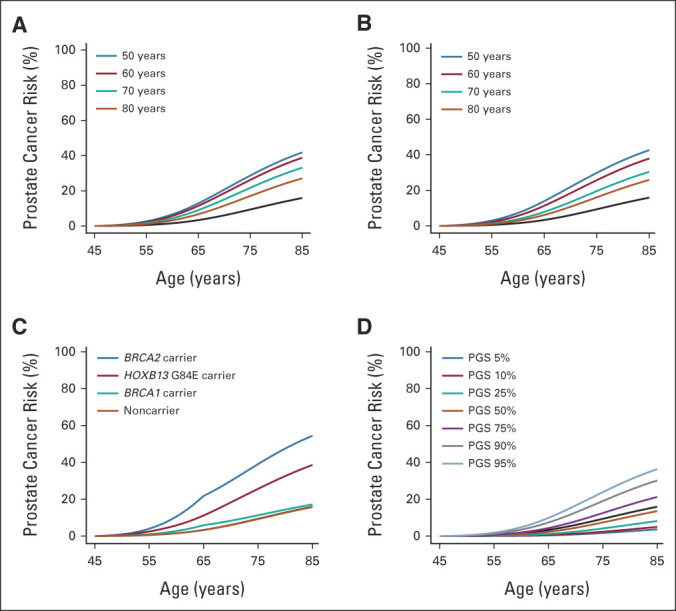
Predicted cumulative prostate cancer risks for a 45-year-old consultand by (A) father's age at prostate cancer diagnosis, (B) brother's age at prostate cancer diagnosis, (C) pathogenic variants, or (D) polygenic score percentile. For comparison, all graphs show the population average risk (black curve). Consultands and brothers were assumed to be born after 1960, and fathers were assumed to be born in the 1930-1939 birth cohort.

**FIG 2.**
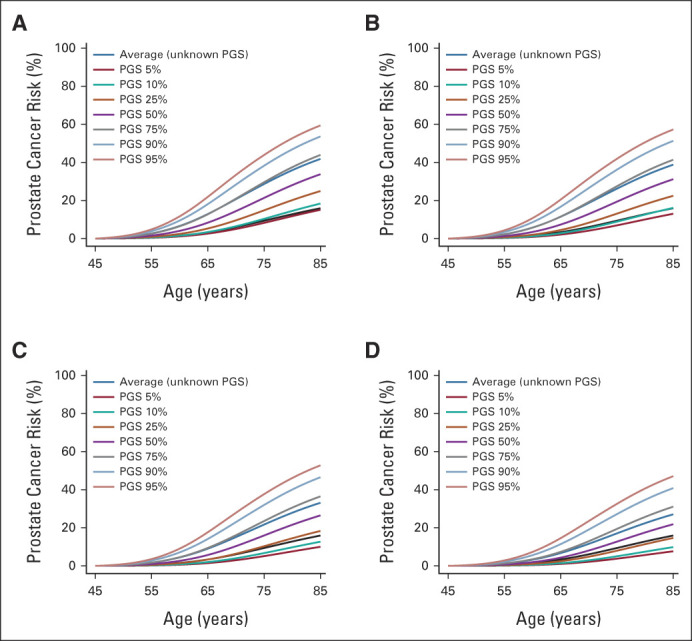
Predicted cumulative prostate cancer risks for a 45-year-old consultand by combinations of family history and PGS percentile: (A) father diagnosed at age 50 years, by polygenic score; (B) father diagnosed at age 60 years, by polygenic score; (C) father diagnosed at age 70 years, by polygenic score; and (D) father diagnosed at age 80 years, by polygenic score. For comparison, all graphs show the population average risk (black curve). Consultands were assumed to be born after 1960 and fathers were assumed to be born in the 1930-1939 birth cohort. PGS, polygenic score.

**FIG 3.**
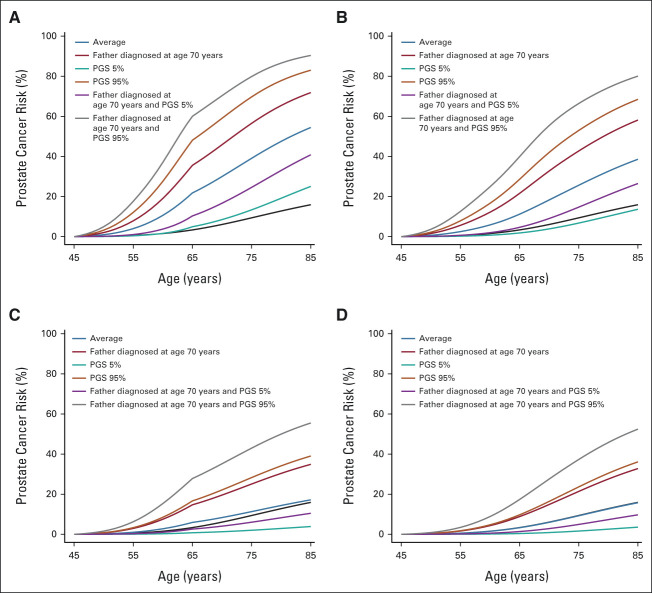
Predicted cumulative prostate cancer risks for a 45-year-old consultand by combinations of family history, pathogenic variant, and PGS percentile: (A) *BRCA2* pathogenic variant carrier, by family history and polygenic score percentile; (B) *HOXB13* G84E carrier, by family history and polygenic score percentile; (C) *BRCA1* pathogenic variant carrier, by family history and polygenic score percentile; and (D) noncarrier of pathogenic variants in *BRCA2*, *HOXB13*, and *BRCA1*, by family history and polygenic score percentile. For comparison, all graphs show the population average risk (black curve). Consultands were assumed to be born after 1960 and fathers were assumed to be born in the 1930-39 birth cohort. PGS, polygenic score.

The average population risk is 16% by age 85 years. The corresponding model-predicted risk is 54% for *BRCA2* carriers, 39% for *HOXB13* G84E carriers, 17% for *BRCA1* carriers and 16% for noncarriers (Fig 1). On the basis of FH alone, the predicted risk for men with a relative diagnosed at age 50 years is 42% when the father is affected and 43% when the brother is affected. These risks reduce to 27% and 26%, respectively, when the relative's age at diagnosis is 80 years (Fig 1). On the basis of the PGS alone, the predicted risk varies between 4% and 36% between the 5th-95th percentiles of the PGS distribution (Fig 1). The absolute risk differences by PGS are larger in those with FH (Fig 2) and those carrying PVs (Fig 3).

### External Validation

The Data Supplement summarizes the inclusion and the characteristics of the UK Biobank participants. The Data Supplement also details the modified 268-SNP PGS used. There were 3,456 incident PCa cases within 5 years and 7,624 within 10 years.

### Discrimination

The predicted risk on the basis of age had a C-index of 0.716 (95% CI, 0.709 to 0.723) for prospective PCa diagnosis within 5 years and 0.693 (95% CI, 0.688 to 0.698) within 10 years. Adding FH, PV, or PGS information increased the C-indices. Including all available information, the C-indices were 0.790 (95% CI, 0.783 to 0.797) and 0.772 (95% CI, 0.768 to 0.777) for predicting 5- and 10-year risks, respectively (Table 2).

**TABLE 2.**
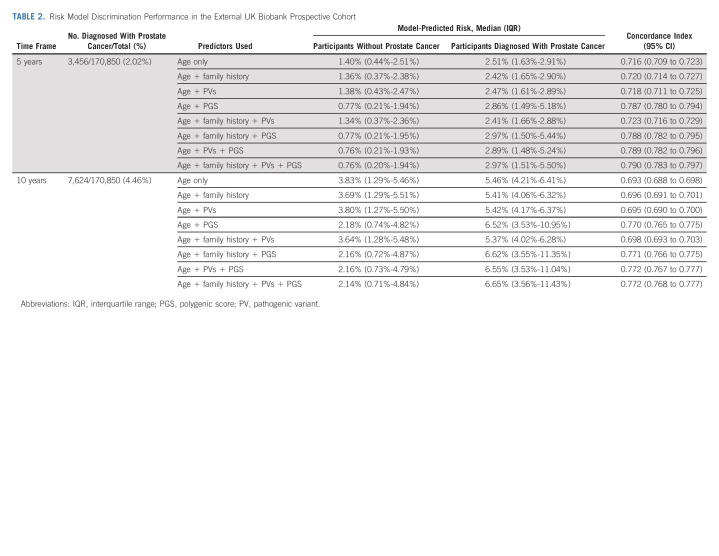
Risk Model Discrimination Performance in the External UK Biobank Prospective Cohort

In subgroups defined by age, FH, PV status, or PGS quartile, the corresponding C-indices ranged between 0.702-0.806 for 5-year and 0.692-0.789 for 10-year risks (Data Supplement).

### Calibration

**FIG 4.**
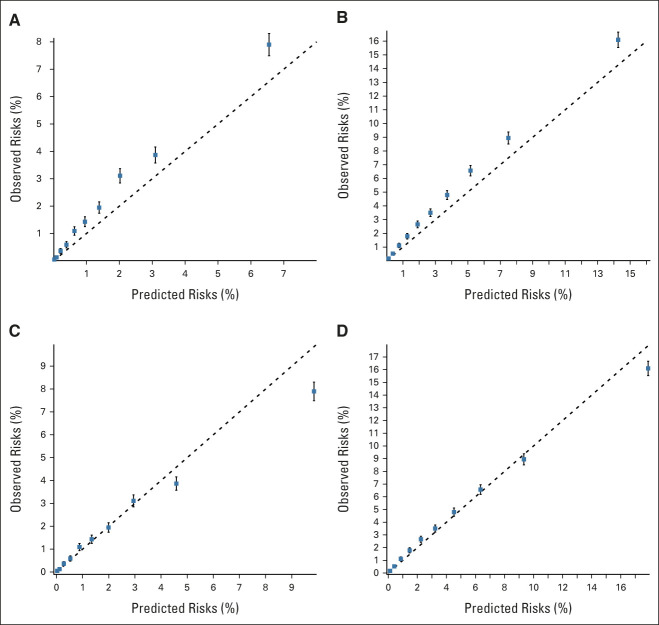
Calibration plots of the model-predicted and observed prostate cancer risks in the external UK Biobank validation cohort. The graphs show the mean predicted risk under the risk model on the basis of all age, family history, pathogenic variant, and polygenic score information available at baseline, within groups defined by the deciles of the model-predicted risks, against the corresponding observed prospective risks on the basis of the Kaplan-Meier estimator: prostate cancer risk within (A) 5 years; (B) 10 years; (C) 5 years, after recalibrating the risks to account for the excess prostate cancer risk observed in the UK Biobank participants; and (D) 10 years, after recalibrating the risks to account for the excess prostate cancer risk observed in the UK Biobank participants (Data Supplement).

The predicted risks on the basis of all available information appeared to systematically underestimate the observed risks (Figs 4A and 4B). The underestimation was however apparent also when based only on the year- and age-specific population incidence (Data Supplement), and in age-, FH-, or PV status–based subgroups (Data Supplement), indicating a higher PCa incidence in UK Biobank participants compared with the UK population incidence. After recalibrating the predicted risks to account for the excess overall risk in UK Biobank (Data Supplement),^57^ the model-predicted and observed risks were generally similar, both in the full data set (Figs 4C and 4D) and in subgroups (Data Supplement). The results indicated that the recalibrated risks might be somewhat overestimated in the highest-risk decile (Figs 4C and 4D), but the difference was small (ratio of observed/predicted 10-year risks = 0.90; 95% CI, 0.87 to 0.93), and in participants with FH (Data Supplement).

### Risk Classification

The participants with the top 1% of the predicted risks included 7.2% and 5.8% of the observed PCa cases within 5 years and 10 years, respectively. Expanding to the top 10% of the predicted risks identified 38.5% and 34.8% of the cases, respectively. 89.1% and 86.3% of the cases, respectively, had above-median predicted risks (Data Supplement).

## DISCUSSION

We have developed a comprehensive genetic PCa risk model for European ancestry men, using UKGPCS, the largest family-based PCa study of its kind. The model allows for personalising PCa risks on the basis of a consultand's age, detailed cancer FH, moderate- to high-risk *BRCA2*, *HOXB13,* and *BRCA1* PVs, and a 268-SNP PGS. In the large independent prospective UK Biobank cohort, the model discriminated well between individuals unaffected or affected with PCa within 5 or 10 years, and the predicted risks were in line with the observed risks after recalibration to accommodate an above-population risk in the cohort.

In the model, familial PCa aggregation is explained by the known PVs, a PGC with a SD that decreases with age, together with an additional high-risk recessive allele. The 268-SNP PGS explains 52.3% (95% CI, 50.3 to 54.4) of the PGC's SD. The putative recessive high-risk allele is consistent with the higher FRRs observed between brothers than in father-son pairs in this study and in previous observational studies.^1,2^ The result is also consistent with previous segregation analysis studies.^58-60^ However, to date, to our knowledge, no PCa recessive susceptibility loci have been identified, and it is more likely that such a recessive component reflects several alleles that collectively behave in a recessive manner, or potentially other factors that explain the FRR patterns. In particular, the patterns might be driven by more frequent PSA testing in brothers than sons of affected men, as men with PCa FH are more likely to be PSA-tested than other men^61^ and PCa FRRs are higher during the first year after a FDR's PCa diagnosis,^62,63^ particularly after a brother's diagnosis.^62^ The estimated RR for homozygote carriers was higher when the method of diagnosis was ignored in the ascertainment adjustment and in the subgroup of families of probands diagnosed by PSA test, indicating that the result may partially be driven by PSA screening effects. However, early reports also suggested higher risks for brothers of affected men than for sons, even before widespread PSA test availability.^64^ In addition, twin studies found that little PCa risk variation is attributable to shared familial nongenetic factors.^3,4^ Taken together, these suggest that variants which act in a recessive manner may explain some of the higher FRR to brothers of cases, but direct identification of such variants in association studies will be required to confirm this. Notwithstanding, the model provides a good fit to the data and hence a rational basis for risk prediction.

In family-based studies, relatives are ascertained through an affected family member and are generally at a higher-than-average risk of disease. Therefore, it is critical to adjust for the ascertainment to avoid biased parameter estimates.^65-67^ The participants diagnosed by PSA testing had FRRs that were higher than FRR estimates reported in population-based studies.^1,2^ This may reflect a greater PSA screening rate by FH.^61^ To address this, we adjusted for potential ascertainment because of family phenotypes in all families of probands who were not diagnosed through symptomatic PCas. This provided FRR estimates that are consistent with those reported in large population-based studies.^1,2^

The PCa risks observed for UK Biobank participants were higher than corresponding year- and age-specific population incidences. The UK Biobank participants have been reported to have higher socioeconomic status than the general UK population.^68^ PSA testing rates vary by socioeconomic status,^69^ and might explain this excess PCa risk. Consistently, the model-predicted risks underestimated those in UK Biobank, but after adjusting for the overall excess PCa risk in the cohort, the predicted risks were consistent with the observed risks in most risk categories.

The model can be expanded with the inclusion of new PVs, as evidence and reliable risk estimates become available for additional genes associated with PCa risk.^45,70-75^ Similarly, although the model incorporates the latest 268-SNP PGS,^18^ the model is flexible and can incorporate alternative PGSs, provided that an estimate of the proportion of the PGC that is explained by the PGS is available.^76^ As further risk variants are identified, the model discrimination is expected to improve.

The validation results demonstrate that the model provides high levels of PCa risk-stratification in the population, and hence might facilitate the identification of men who could benefit from screening and other early detection interventions. For example, the half with above-median predicted risks included 89.1% of all prospective PCa cases observed within 5 years. Previous research has suggested that targeted PSA-based screening of *BRCA2* PV carriers^8,77^ or on the basis of PGS stratification could reduce overdiagnosis rates^78^ and be cost-effective.^79^ Future studies should evaluate the impact of risk-stratified screening on the basis of a more comprehensive risk prediction model such as the model presented here.

The study has limitations. The ascertainment adjustment is limited by a lack of data on PSA testing history in the UKGPCS families and data on whether FH influenced screening decisions of PSA-test–diagnosed probands; it may be an overadjustment that has resulted in reduced precision in the parameter estimates compared with the estimates that could have been achievable if exact information were available. A growing body of evidence suggests that the risk to *BRCA2* carriers varies by the location of the PV within the gene.^80-82^ The model does not incorporate this variation. This requires more precise estimates of the risks associated with PVs in each region than are currently available. The use of self-reported cancer FH data may be limited by under-reporting and inaccuracies.^83^ However, model-predicted FRRs were consistent with FRRs reported in observational studies. Furthermore, the participants were unaware of their genotypic information at study entry, and so, differential reporting of FH by PV status or PGS is unlikely. In the validation cohort, the FH data did not include information on relatives' age at diagnosis or information on unaffected relatives. We inferred plausible ages at diagnosis on the basis of assumed familial age structures, but did not make assumptions about the unaffected relatives. This may explain the somewhat higher-than-expected risks in the FH-positive subgroup, as inclusion of unaffected relatives would have attenuated the risks. Despite these limitations, there was a clear gradient toward higher observed risks with higher predicted risks, and the predicted risks discriminated well between cases and noncases also in the subgroup with FH. *BRCA2* PVs are associated with high-grade PCa,^5,8,9^ but previous evidence suggests that overall risks on the basis of *HOXB13*^11-13^ or *BRCA1* PVs^5,8,9^ or the 268-SNP PGS^25^ are similarly predictive of high-/low-grade PCa. Both UKGPCS and UK Biobank lacked grade data on the self-reported PCas in relatives, so we could not estimate grade-specific FRRs, despite some previous observational evidence suggesting that brothers tend to develop similar-grade PCas.^84^ Grade data on UKBiobank participants' incident PCas are not currently available; therefore, validation of grade-specific risks was also not possible. However, the majority of the UKGPCS probands had symptomatic PCas, which tend to be more aggressive than preclinical PCas.^85^ Taken together with the *BRCA2* risks^5,8,9^ and evidence suggesting grade-specific FRRs,^84^ it is likely that the model predictions reflect more clinically significant disease risks. This may also partly explain the underpredicted risks in UK Biobank, before recalibration. However, further research is needed on genetic predictors for aggressive PCa and on validating the prediction of specifically aggressive PCa risks. The model does not incorporate nonfamilial/nongenetic factors, such as PSA or other clinical measurements. Importantly, the model was developed and validated in men of European ancestry. PCa risks are higher in men of African ancestry and lower in men of Asian ancestry,^86^ and further adaptation will be required to provide calibrated risks across all ancestries.

In conclusion, to our knowledge, this multifactorial risk prediction model is the first to incorporate the effects of the currently known moderate- to high-risk and common low-risk PCa risk variants together with detailed FH information. The model predicts consistent familial risks and shows good discrimination and calibration in an independent prospective validation cohort. The model will be beneficial for counselling of men in cancer family clinics, and can form the basis for future research evaluating risk-stratified population screening approaches.

## Data Availability

Individual pedigree-level data from UKGPCS are not publicly available as individuals could potentially be identifiable from the family structure. However, we confirm that summary-level data are available on request. The data that were used for validation are available by application to UK Biobank (https://www.ukbiobank.ac.uk/enable-your-research). Sufficient information on the risk prediction algorithm and on the genetic and familial predictive components to allow replication is provided in the manuscript and the Data Supplement. The algorithms are also available on request for research purposes from the authors.
